# Combination of Simultaneous Artificial Sensory Percepts to Identify Prosthetic Hand Postures: A Case Study

**DOI:** 10.1038/s41598-020-62970-4

**Published:** 2020-04-20

**Authors:** Jacob L. Segil, Ivana Cuberovic, Emily L. Graczyk, Richard F. ff. Weir, Dustin Tyler

**Affiliations:** 1 Rocky Mountain Regional VA Medical Center, Rehabilitation Research and Development, Denver, CO 80220 USA; 2 University of Colorado Boulder, Engineering Plus Program, Boulder, CO 80309 USA; 3 Case Western Reserve University, Department of Biomedical Engineering, Cleveland, OH 44106 USA; 4 Louis Stokes Cleveland Veterans Affairs Medical Center, Cleveland, OH 44106 USA; 5 University of Colorado Denver|Anschutz Medical Campus, Department of Bioengineering, Aurora, CO 80045 USA

**Keywords:** Sensory processing, Translational research, Biomedical engineering

## Abstract

Multiple sources of sensory information are combined to develop hand posture percepts in the intact system, but the combination of multiple artificial somatosensory percepts by human prosthesis users has not been studied. Here, we report on a case study in which a person with transradial amputation identified prosthetic hand postures using artificial somatosensory feedback. He successfully combined five artificial somatosensory percepts to achieve above-chance performance of 95.0% and 75.7% in identifying four and seven postures, respectively. We studied how artificial somatosensation and the extant hand representation are combined in the decision-making process by providing two mappings between the prosthetic sensor and the location of the sensory percept: (1) congruent, and (2) incongruent. The participant’s ability to combine and engage with the sensory feedback significantly differed between the two conditions. The participant was only able to successfully generalize prior knowledge to novel postures in the congruent mapping. Further, he learned postures more accurately and quickly in the congruent mapping. Finally, he developed an understanding of the relationships between postures in the congruent mapping instead of simply memorizing each individual posture. These experimental results are corroborated by a Bayesian decision-making model which tracked the participant’s learning.

## Introduction

Humans have evolved specialized hands and complex neural systems that enable us to skillfully use a multitude of tools^1–7^. In order to grasp and use these tools, we adopt hand postures that are appropriate for the size, shape, and intended manipulation of each tool^5,7–10^. For example, people use cylindrical grasps to pick up cups but use lateral grasps to turn a keys in locks. Each grasp involves precise, coordinated control of several limb segments and is associated with distinct patterns of somatosensation including proprioception and tactile feedback from the grasped object^7^. This somatosensory feedback is transduced by several types of mechanoreceptors in the skin, joints, and muscles, then processed in the spinal cord and cortex to form a holistic percept of the grasp^11^. Successful tool use thus requires both the ability to position the hand into specific grasp postures and appropriate feedback from the hand about the attained posture^7^.

Amputation of the hand leads to mechanical and sensory deficits in grasping^12–14^. Persons with upper limb loss typically rely on prosthetic hands to be able to hold and manipulate objects or perform tasks. However, current prosthetic technologies do not provide the components necessary for effective tool use. To use tools skillfully, prostheses require the mechanical ability to achieve different postures, use of intuitive control schemes, and provide somatosensory feedback of posture and object interactions to the user. Towards the creation of mechanical prosthetic components, dexterous fingers and wrists have been well studied^15–17^. In fact, several prosthetics research groups and commercial companies have developed prostheses with multiple grasp options, such as the Otto Bock beBionic and Michelangelo, the TASKA hand, and Mobius LUKE arm. The feedforward control of advanced prosthetic components to provide intuitive myoelectric control has also progressed dramatically^18–21^, and control schemes for multiple degrees of freedom are also commercially available, including Complete Control from CoApt, Chicago IL, Myo Plus from Otto Bock, Duderstadt, Germany, and Sense from Infinite Biomedical Technologies, Baltimore, MD.

While the mechanics and control strategies for prostheses have advanced rapidly in recent years, current commercial prostheses do not restore the sensory information formerly provided by the hand. This lack of somatosensory feedback forces users to rely other sources of feedback, such as vision, to regulate movements^22,23^, likely limiting their ability to perform tasks that divert visual attention from the prosthetic device while it is in use. Indeed, many users state a desire for improved sensory feedback^22–24^, and they rarely use prostheses for complex motor tasks such as delicate tool manipulation. Instead, they use prostheses for supporting or bracing tasks, use them as passive devices, or simply do not use them^23–25^. Numerous groups are developing electrode technologies to provide artificial sensory feedback to amputees by electrically activating the residual somatosensory nerves of the hand^26–28^. Our laboratory has shown that electrical stimulation can elicit tactile and proprioceptive sensations that are located on the hand and are scalable in modality and intensity^26,29,30^. Prior studies have also examined the impact of artificial somatosensation on functional tasks such as object identification^31^, object feature discrimination^28,32–35^, and closed-loop control^36,37^. Unsurprisingly, these studies showed improvement in functional task performance when somatosensory feedback was provided to the prosthesis user as opposed to when it was not.

While many of these tasks provided participants with multiple sensory feedback percepts, they were possible to execute by distinguishing gradations of a single percept. For instance, proprioceptive feedback of hand aperture can be used to determine object size^35^, and intensity can be used to determine whether an object is being contacted^31^. The extent to which multiple sources of artificial sensory information are combined with each other to develop a single, holistic percept like prosthetic hand posture has not been studied. A person must identify a posture in realistic settings by considering both the entirety of the sensory information presented and the goals of the task. In the intact system, multiple sources of somatosensory information are combined in order to provide unified perception of objects or grasping interactions^38–41^. This process of combining information varies as a function of the available information^41–43^, information complexity^44^, and the existence or applicability of prior knowledge during functional tasks^44–46^. Further, the manner and extent of information combination can vary as the task is either learned or the provided information changes^41,43,47–50^. Much of the literature on intact sensorimotor control indicates that this sensory fusion is often a subconscious process whose output is a conscious decision^47,51–57^.

Bayesian mathematics^47,56–61^ provides a framework for studying changes in decision-making resulting from learning^48–50^ or varying sensory feedback^41,47^. This statistical method combines uncertain measurements based on their relative confidence so as to improve the overall certainty of the combined measurement. When applied to decision making, this principle ensures that the total number of correct decisions is maximized across time. Further, the relative weighting for input is updated as information is learned or the relative certainty of a particular sensor changes. Thus, the flexibility of this model makes it well-suited to modeling learning or combinatorial processes, which are known to be dynamic^62,63^.

Here, we present a case study in which we study the ability of a person with amputation to combine multiple simultaneous, electrically-evoked sensory percepts and use this combination to identify multi-functional prosthetic hand postures. We define combination as the merging of information from multiple, unimodal somatosensory percepts into a single, holistic percept. We investigate how training affects the participant’s ability to use artificial somatosensation and how learned knowledge is applied in novel tasks. We study how artificial somatosensation and the hand mapping in the extant hand representation are combined by varying the alignment between each prosthetic sensor and the location of the sensory percept it evokes. We hypothesize that the participant can combine five unimodal, but distinct artificial somatosensory percepts to identify multiple complex hand postures. We further hypothesize that his ability to combine percepts will be improved when sensory feedback is provided in a manner that is congruent with his extant hand representation.

## Methods

### Participant

In this case study, the participant is a 43-year old male who sustained a trans-radial amputation 74 months prior to this study. He was implanted with two 16-channel Composite Flat Interface Nerve Electrodes^64^ proximal to the elbow on his median and ulnar nerves. All data was collected during two 5-hour experimental sessions 17 months after implant. All study devices and procedures were reviewed and approved by the U.S. Food and Drug Administration Investigational Device Exemption, the Cleveland Department of Veterans Affairs Medical Center Institutional Review Board, and the Department of the Navy Human Research Protection Program. All study procedures and experiments were performed in accordance with relevant guidelines and regulations of these institutions. Written informed consent was obtained from the subject.

### Experimental setup and peripheral nerve stimulation

**Figure 1:**
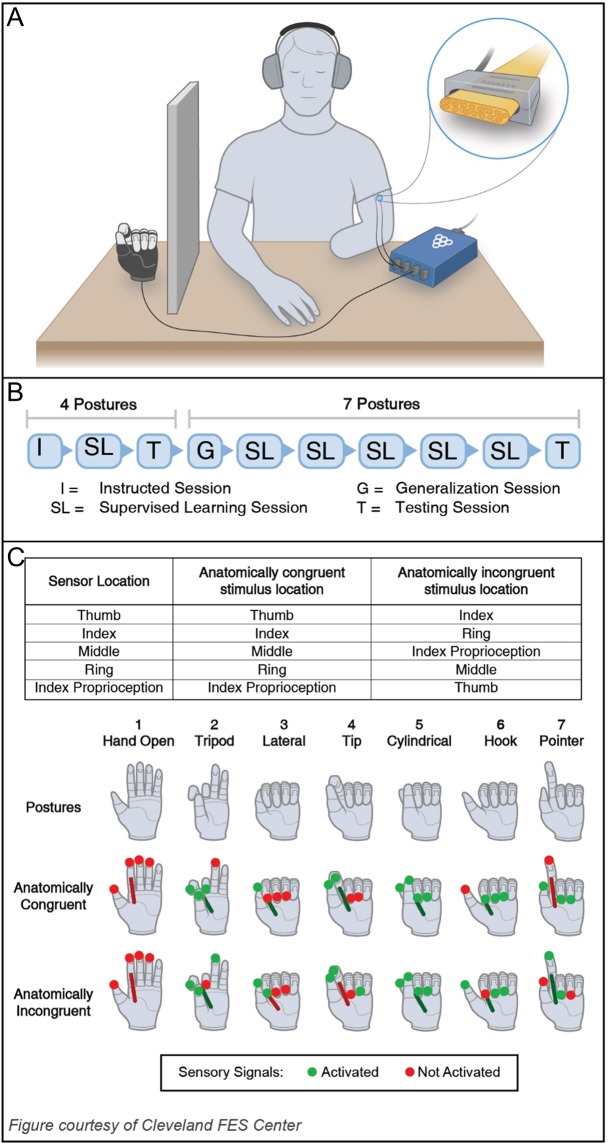
(**A**) The experimental setup using the TASKA prosthetic hand, the Grapevine Neural Interface Processor, and the implanted electrodes in the residual limb of the participant. (**B**) Table: The prosthesis sensor locations and the location of the corresponding evoked percepts for the congruent and incongruent mappings. Infographic: the active prosthesis sensors for each postures (top); the locations of the evoked percepts associated with the posture in the congruent (middle) and incongruent (bottom) mappings. (**C**) The experimental procedure sequence included I, SL, T, and G blocks in which either four or seven postures were presented.

Electrical stimulation of the participant’s nerves was controlled by a custom Matlab (MathWorks Inc.) graphical user interface (GUI) and enacted through the Grapevine Neural Interface Processor (Ripple LLC). Stimulation consisted of biphasic, cathode-first, charge-balanced pulse trains. All stimulation parameters were held constant for the duration of the experimental session. Pulse frequency and width were set to 100 Hz and 250 μs, respectively. Pulse amplitudes for each contact were adjusted such that the participant verbally reported on a self-defined, open-ended scale that all evoked percepts were of similar intensities and that each individual percept was both detectible and comfortable when all sensors were active. Stimulation pulses for each independent sensory percept was interleaved to ensure that stimulation pulses were asynchronous, but perceived sensations were synchronous. The participant entered their responses and controlled trial progression through a Wacom® tablet PC monitor (Cintiq 27QHD, Wacom International, USA). A TASKA prosthetic hand (TASKA Prosthetics, Inc.) with custom fingertip sensors^65^ (Fig. 1A) was commanded into various postures to provide visual feedback using a custom LabView (National Instruments, Inc.) interface.

### Experimental procedure

The participant engaged in a forced-choice posture identification task based on sensory feedback. Both an abbreviated four-posture task and an expanded seven-posture task were implemented to represent simple and a difficult identification tasks, respectively^66^. The seven functional postures^3,9,67^ included (1) hand open, (2) tripod, (3) lateral prehension, (4) tip prehension, (5) cylindrical prehension, (6) hook, and (7) pointer (Fig. 1B, top). The four-posture task included postures 1–4 only.

Combinations of tactile sensation on four fingertips and proprioception of the ring finger provided feedback for each posture (Supplemental Table 1). Sensation locations and modalities were chosen to provide feedback with unique and describable percepts. The participant was told that sensation was driven by interactions with the prosthesis sensor. However, he was actually provided with pre-recorded stimulation patterns to ensure consist feedback and to eliminate other potential sources of feedback. He also wore noise-cancelling headphones to eliminate auditory feedback.

Each experimental session consisted of ten experimental blocks. The participant was instructed to identify the presented posture as quickly and accurately as possible using the provided feedback (Table 1). Four block types were implemented. During the instructional block (I, Fig. 1C), a four-posture menu was introduced, and the participant received temporally-coordinated visual and sensory feedback for each posture; he could both see which posture the hand was in and feel which sensors were contacting one another. This block was the only one in which visual feedback was provided. As this block was only intended to instruct the subject on the experimental protocol, data from this block was excluded from experimental analysis. For the remaining blocks, visual feedback was eliminated: the prosthesis was placed behind a barrier. The supervised learning (SL) blocks were intended to train the participant to use only provided somatosensation to identify postures. In these blocks, the participant received sensory feedback for each posture. The correct response was visually displayed through the GUI after each trial. The generalization (G) block evaluated the participant’s ability to extrapolate existing knowledge to novel stimuli. Here, the full seven-posture menu with three novel postures was introduced. The participant was not provided with information about the postures prior to this block and did not receive corrections to his responses during the block. Finally, during the testing (T) blocks, he was evaluated on his retention and asked to identify the previously learned postures without any corrections to his responses. Every posture was presented ten times in a randomized fashion in each block. Further, the same sequence of I, SL, T, and G blocks (Fig. 1C) was implemented each session to enable comparisons between sessions. After each block, the participant was asked to verbally describe his strategy for identifying postures.

**Table 1:** Provided feedback in each testing block type.

Block Type	Visual Feedback	Tactile Feedback	Corrections Provided	Novel Postures Introduced
Instructional	✓	✓	✓	✓
Supervised Learning		✓	✓	
Generalization		✓		✓
Testing		✓		

Visual feedback was defined as watching the TASKA hand move to the tested posture. Sensory feedback was defined as artificial somatosensation triggered by contact with pressure sensors on the TASKA fingertips or by flexion of the TASKA index finger. Corrections were defined as clarifying the tested posture to the participant immediately after each trial. Novel postures introduced refers to the first time that previously untrained postures were tested in an experimental block.

Two mappings between the locations of the sensors on the prosthetic hand and the locations of the evoked sensory percepts were tested (Fig. 1B). The active prosthesis sensors for each posture remained consistent between the mappings; only the locations of the corresponding evoked sensory percepts changed. In the congruent mapping, the location and modality of the tactile sensory percepts were aligned with the prosthesis sensors, and the modality of the proprioceptive sensory percept was aligned with is prosthesis sensor. For example, pressure on the prosthesis index finger sensor triggered a tactile sensation on the participant’s index finger. Only the percept modality, instead of both location and modality, was aligned for the proprioceptive sensor because the participant has limited channels that reliably evoke proprioception. In effect, the proprioceptive sensation was incongruent in its spatial mapping during all experimental conditions. Evoked proprioception of the ring finger corresponded to flexion of the prosthesis index metacarpophalangeal joint. In contrast, the location of evoked sensations was not aligned with the prosthesis sensors in the incongruent mapping. The requirements for the incongruent mapping included: (1) the prosthesis sensor location and its corresponding evoked sensory location cannot be the same, and (2) the incongruent mapping for any given posture cannot match the congruent mapping of any other posture. For example, evoked proprioception of the ring finger provided information about contact with the prosthesis middle finger sensor. The incongruent mapping stayed constant throughout the experimental session, so the participant could learn the mapping over time. The two mappings were tested on separate days to ensure that performance in one would not influence performance in the other. Furthermore, other experiments that did not rely on the incongruent mapping took place between the two sessions to promote “wash-out” of the previously trained mapping.

### Bayesian modeling

While the experimental results can track the participant’s responses, they do not provide insight into the decision making process. Post-hoc, we constructed a Bayesian decision model to gain insight as to how the subject might be incorporating prior information in subsequent decisions. Specifically, we asked the question as to whether or not prior information is used differently in the congruent versus incongruent conditions.

In Bayesian mathematics, a “prior” and a “likelihood” are combined to determine performance on the current task, or “posterior”^57^ (Eq. 1). The prior encodes all previous understanding of the task, while the likelihood defines the task itself. Here, we defined the prior, *p*(*x*), to be the discrete probability distribution function that describes the participant’s response when presented with a specific posture. The Bayesian prior for trial 1 of each block was defined as the confusion matrix of the participant’s experimental performance from the preceding block and was updated following each trial. This update was implemented three ways to query different types of participant learning. The “no update” implementation maintained a constant prior throughout the block. In the “experimental” implementation, the prior was recalculated after each trial using the participant’s experimental response, regardless of the presented posture. For instance, if the participant responded with posture 3 (tripod) when posture 5 (cylindrical) was presented, the prior for posture 5 would be updated to reflect an increased probability of responding incorrectly with posture 3 the next time posture 5 was presented. Finally, the “presented” implementation, the prior was recalculated after each trial using the actually presented posture regardless of participant response. Using the same example as above, the prior for posture 5 would be updated to reflect an increased probability of responding correctly to posture 5.

We defined the likelihood, *p*(*o*|*x*), as the probability of a particular posture being presented in a trial. As all seven postures were presented an equal number of times, the likelihood is the uniform probability distribution at 14%. Gaussian noise with magnitude of ±7% was added to depict sensorimotor noise inherent to physiological systems^68^. The magnitude of the Gaussian noise was chosen to elicit variability in the final modeled decision.

The Bayesian posterior, *p*(*x*|*o*), was calculated as the dot product of the prior and likelihood distributions (Eq. 1). The outcome with the maximum value of the posterior probability density function was selected as the Bayesian response for that trial^54^. As this model contains noise, the Monte Carlo method was used to determine average model behavior. Each block was simulated 100 times under each update rule. The model’s accuracy in identifying postures was determined for each iteration, and the percent error of the model was calculated by comparing the model’s identification accuracy to the participant’s identification accuracy in the analogous experimental block (Eq. 2).

1 $$p(x|o)=\frac{p(x)p(o|x)}{p(o)}$$

2 $$Percent\,Error=\frac{{\sum }_{i=1}^{100}||Modeled\,Accurac{y}_{i}-Measured\,Accuracy||}{100}$$

### Experimental results analysis

The participant’s response and response time were recorded for each trial. Accuracy, a direct indicator of the participant’s ability to complete the task, was determined by comparing the participant’s response to the presented posture. Response time, an indirect indicator of cognitive effort^69–73^, was defined as the duration of time between stimulation ending and the participant responding. The timestamps for trial start, stimulation end, and participant response were automatically collected in Matlab. After each block, the participant was asked to verbally describe his strategy for identifying postures. All responses were captured in video recording for later analysis.

Inherent differences between postures were evaluated. One such difference is the amount of exposure to the posture the participant received. Postures were classified as either “trained” or “novel” in both mappings. Postures 1–4 (“trained” were presented in all ten blocks. Postures 5–7 (“novel”) were only presented in blocks 3–10.

Another difference between postures is how similar or dissimilar they are to one another. Hamming distances^74^ quantify the similarity between pairs of postures as a function of active prosthesis sensors. Here, the Hamming distance between two postures describes the number of sensors that differed in activation between the two postures. A small Hamming distance indicates that feedback for the two postures was similar. For example, the cylindrical and hook postures only differ in activation of the thumb sensor, so the Hamming distance between them is one. However, the hook and lateral postures differ in activation of the thumb, index, middle, and ring finger sensors, so the Hamming distance between them is four.

### Statistical analysis

Statistical analyses were performed using Minitab Statistical Software (Minitab, Inc.). Accuracy and response-time were analyzed using tests of 1- and 2- proportions and t-tests, respectively. The specific test for each comparison is described in Results below. The Bayesian model outputs were analyzed using a 1-way ANOVA followed by a Tukey test. Significance was calculated using an alpha value of 0.05 throughout. All data are reported as the mean ± the standard error of the mean.

## Results

### Hand postures can be identified using multi-modal sensory feedback

**Figure 2:**
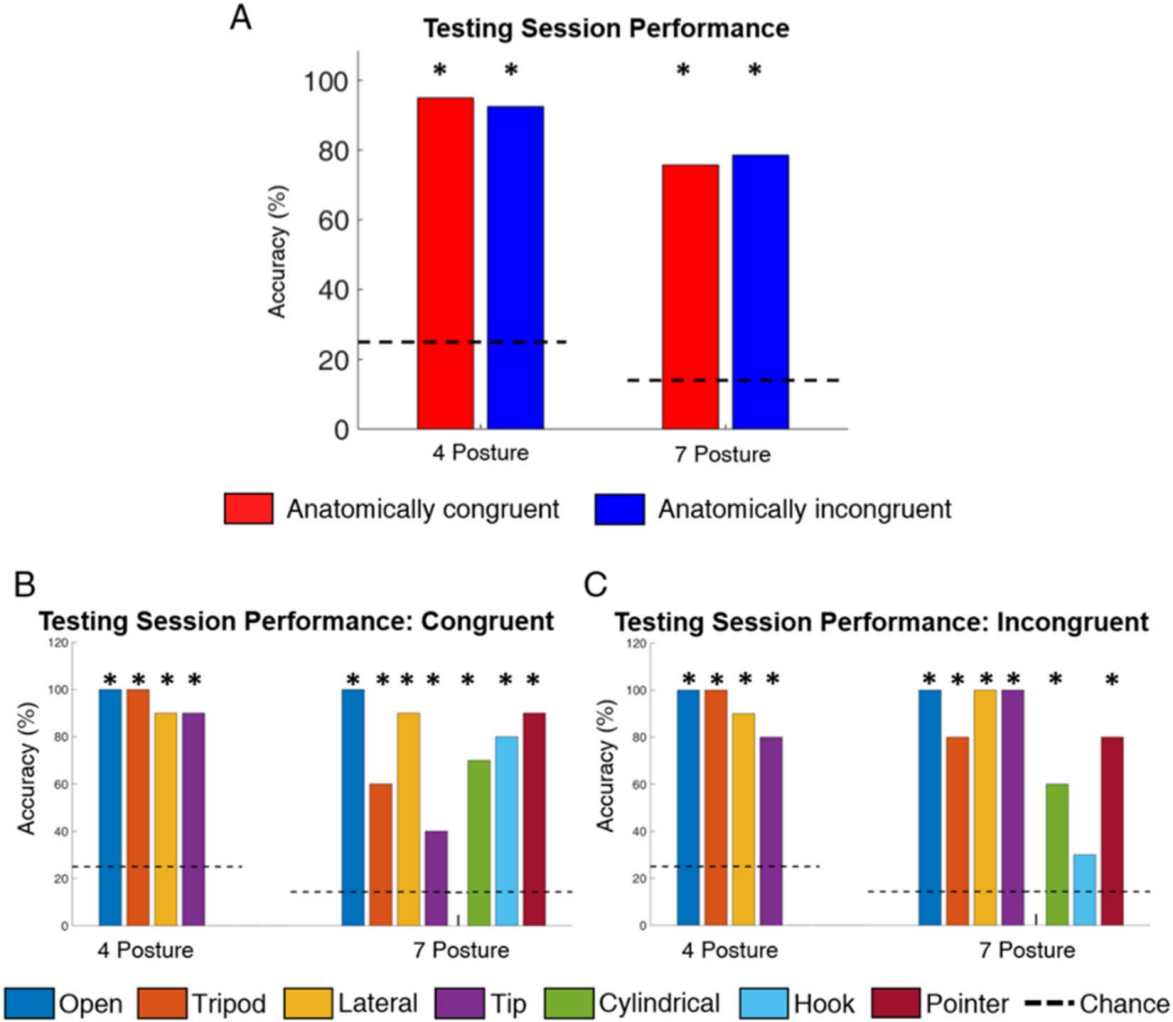
(**A**) Overall accuracy in identifying postures in the four- and seven-posture T blocks. There were no significant differences in overall accuracy between the congruent and incongruent mappings during these blocks. In all cases, the subject performed significantly better than chance as indicated by the asterisks (n = 40, 70 trials in the four- and seven- posture blocks, respectively). (**B,C**) Accuracy in identifying individual hand postures during T blocks in the congruent (**B**) and incongruent (**C**) mappings. In all cases except incongruent hook, identification accuracy was significantly above chance, as indicated by the asterisks (n = 10 trials per posture).

We verified in the T session that the identification of hand postures using only artificial somatosensory feedback is possible. After five SL blocks, the participant was able to develop a strategy for each of the seven hand postures for both the congruent and incongruent mappings. Both strategies were sufficient to achieve a global accuracy significantly above chance during the T block (Fig. 2A). In the congruent case, the participant’s average accuracy during the T block across four and seven postures was 95.0% and 75.7%, respectively. Similarly, his performance in the incongruent mapping was 92.5% and 78.6%.

The participant was able to identify postures (tests of 1-proportion, *p* < 0.05) using only the provided sensory feedback. His performance in differentiating postures in the four-posture T block ranged between 80 and 100% for both congruent and incongruent mappings. He had difficultly, however, in identifying specific postures in the 7-posture T block. The differences in erroneous postures identification between congruent and incongruent mapping offers insight into the subjects use of available information (Fig. 2B,C). In the congruent mapping, the participant was able to identify all novel postures significantly above chance performance (tests of 1-proportion, p < 0.001) but had diminished performance in two of the trained postures (tripod and tip, Fig. 2B). In contrast, the participant perfectly identified three of the trained postures but performed poorly on the novel postures Fig. 2C) in the incongruent mapping. In fact, he was not able to identify “hook” posture significantly above chance performance (test of 1-proportion, p = 0.16). Interestingly, he had difficulty identifying postures that included proprioceptive feedback in both the congruent and incongruent conditions. The participant identified postures that included proprioceptive feedback with an accuracy of 68% and those that did not include proprioceptive feedback with an accuracy of 95% (test of 2-proportions, p = 0.001) in the congruent mapping. Similarly, the participant achieved accuracies 62.5% and 100% (test of 2-proportions, p < 0.001), respectively, when identifying postures with and without proprioceptive feedback in the incongruent condition.

### Generalization of novel postures depends on congruency of information

**Figure 3:**
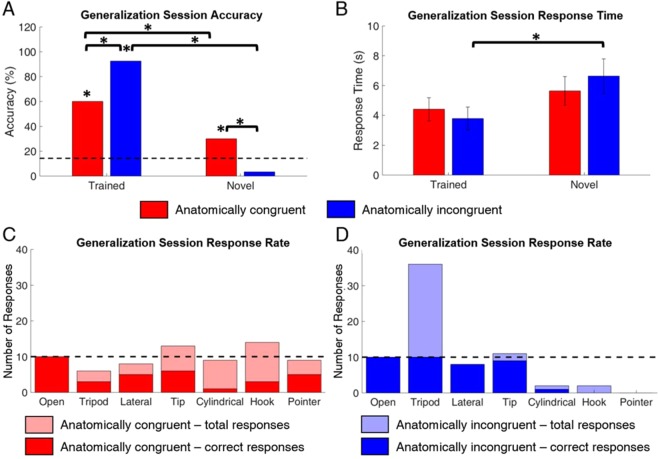
Comparisons between the trained and novel postures during the generalization session are provided here. (**A**) Accuracy of trained versus novel postures in G block across both mappings. Significant differences were seen across the trained and novel postures as well as across congruent and incongruent mappings (n = 40, 30 for trained and novel postures, respectively). (**B**) Mean response time of trained versus novel postures in G block across both mappings. The response time for novel postures was significantly slower than the response time for trained postures in the incongruent mapping (n = 40, 30 for trained and novel postures, respectively). (**C, D**) Total number of responses (light) and number of correct responses (dark) for each posture using congruent (**C**) and incongruent (**D**) mapping during the G block. Note the irregular number of responses across all postures during the anatomically incongruent generalization session and the relative stable number of responses across all postures in the anatomically congruent generalization session (n = 10 presentations per posture).

The participant was able to identify the trained postures significantly more accurately than novel postures during the G block in both experimental sessions (tests of 2-proportions, p = 0.009 and p < 0.001, respectively). A detailed analysis of the accuracy (Fig. 3A) and response times (Fig. 3B) for the trained versus novel postures indicates that he engaged with the trained and novel postures differently in the two mappings. He was only able to generalize knowledge of the trained postures to the novel postures in the G block when provided with sensory feedback in the congruent mapping. He was able to identify novel postures significantly above chance in the congruent mapping (test of 1-proportion, p = 0.029). Similarly, his response time when presented with trained versus novel postures did not significantly differ between trained and novel in the congruent mapping (2-sample t-test, p = 0.439). However, this strategy came at the cost of decreased performance in identifying the trained postures. In contrast, the participant maintained higher performance of the trained postures in the incongruent mapping compared to the congruent mapping: 92.5% versus 60.0%, respectively (test of 2-proportions, p < 0.001). However, he correctly identified only 3.3% of novel postures, a significantly lower accuracy than in the congruent mapping (test of 2-proportions, p = 0.003). He also responded significantly more slowly when presented a novel posture as compared to a trained postures in the incongruent mapping (2-sample t-test, p = 0.002). (Fig. 3B).

The total number of responses for each posture in the congruent (Fig. 3C) and incongruent (Fig. 3D) mapping further highlights differences in the participant’s willingness to attempt identification of novel postures. If the participant fully engaged with all seven postures, then the frequency of responding with any given posture would be uniform Fig. 3C, D, dashed line). However, if he was not engaging with the novel postures, then the trained postures would be over-reported and the novel postures would be under-reported, resulting in a larger variance about the idealized number of responses. In the congruent mapping, the participant immediately engaged with the novel postures and reported all seven postures uniformly (Fig. 3C, light red). The average error between the ideal and reported response rate across postures was only 2.1 ± 0.6 responses. While the participant engaged with all seven postures immediately, he was not accurate in identifying them (Fig. 3C, dark red). In contrast, the participant did not try to identify any of the novel postures in the incongruent mapping, stating “I stuck with the four [postures] I knew.” The average error between the ideal and reported number of responses across posture was 7.8 ± 3.4 responses in the incongruent mapping. This error resulted from a large over-reporting of the trained postures and an associated under- reporting of the novel postures (Fig. 3D, light blue). Specifically, the participant usually responded with “tripod” when a novel posture was presented (Supplemental Fig. 1). As a result, he answered “tripod” 36 times, a 26 response increase over the idealized rate, while only responding with any of the novel postures a total of four times. However, by not engaging with the novel postures, the participant was able to maintain high accuracy of each of the trained postures (Fig. 3D, dark blue).

### Learning through active training improves posture identification

**Figure 4:**
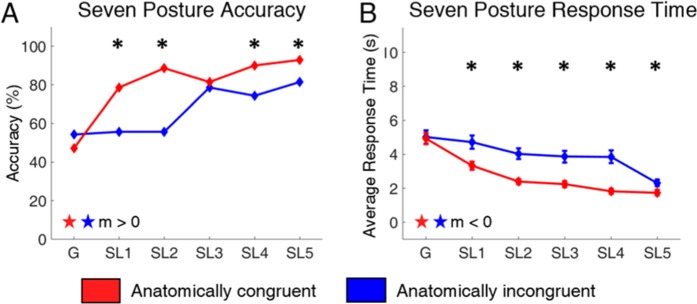
Accuracy (**A**) and Response Time (**B**) for the seven-posture posture identification task from the G to the SL blocks. Incongruent and congruent results show a statistically significant difference across multiple SL blocks. In nearly all sessions, the anatomically congruent mapping causes higher accuracies and faster response times. In both cases, the subject’s performance improved across the G and SL blocks (n = 70 trials per experimental block). The stars indicate the line slope test was statistically significant for both the congruent and incongruent conditions.

We examined if posture identification performance could improve with training by analyzing the participant’s performance in the seven-posture blocks (Fig. 4). While the participant’s initial performance in the G block was poor, he was able to significantly improve his response accuracy (line slope test, p = 0.012 and p = 0.008 for congruent and incongruent, respectively) and decrease his response time (line slope test, p = 0.05 and p = 0.015) with training in the SL blocks. This improvement occurred in both mappings (Fig. 4). In the congruent mapping, accuracy improved from 47.1% to 92.9% (test of 2-proportions, p-value <0.001), and response time decreased from 4.9 s to 1.7 s (2-sample t-test, p-value <0.001). Similarly, in the incongruent mapping, accuracy improved from 54.3% to 81.4% (test of 2-proportions, p-value <0.001), and response time decreased from 5.0 s to 2.3 s (2-sample t-test, p-value <0.001).

### Performance on novel postures depends on congruency of information

Throughout the SL blocks, the participant was able to achieve better outcomes with less cognitive effort when provided with congruent sensory feedback as opposed to incongruent feedback. His accuracy was significantly higher in four of five blocks (Fig. 4A) and response time was significantly lower (Fig. 4B) in all five blocks when using the congruent mapping (p-values ≤ 0.04).

**Figure 5:**
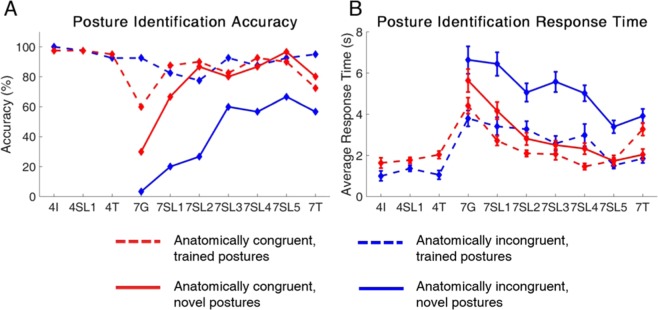
Accuracy (**A**) and Response Time (**B**) for the congruent (red) and incongruent (blue) mappings, distinguished by trained (dashed) versus novel (solid) postures. The performance of the subject show differences between the mapping conditions especially during the G and T sessions. Furthermore, the ability to identify novel postures using the anatomically incongruent mapping was different compared to the trained postures using the anatomically incongruent mapping as well as both the trained and novel postures using the anatomically congruent mapping (n = 40, 30 trials per experimental block for the trained and novel postures, respectively).

There were differences between the two mappings in how the participant integrated the novel postures into his decision-making process during for the SL and T blocks (Fig. 5). The participant immediately began identifying the novel postures in the congruent mapping. His performance was not significantly different (6.9 ± 5.2%, paired t-test, p = 0.236) in identifying novel or trained (Fig. 5A, red lines). Conversely, his ability to identify the novel postures was significantly lower than the trained postures in the incongruent mapping. His accuracy in identifying novel postures was 47.1 ± 8.5% lower than in the trained postures across blocks (paired t-test, p = 0.001) (Fig. 5, blue lines). Indeed, the participant consistently reported difficulty with the incongruent task and described how he was “confused” and needed to “guess” in order to perform the task. However, despite his reported difficulty with the task, his ability to recognize novel postures improved (line slope test, p = 0.006). Further, he maintained high ability to recognize the trained postures. In fact, his performance on those postures showed no significant variation over time through any of the blocks (Fig. 5A, dashed blue line) (line slope test, p = 0.404).

These trends were the same in the cognitive effort exerted by the participant to perform the identification task, as measured by the participant’s response time (Fig. 5B). The participant did not take significantly longer to identify novel postures as opposed to trained postures in the congruent mapping (ΔRT = 0.5 ± 0.3 s, paired t-test, p= 0.198). Conversely, he took 2.4 ± 0.2 s longer to identify novel postures compared to the trained postures in the incongruent mapping (1-sample t-test, p < 0.001).

**Figure 6:**
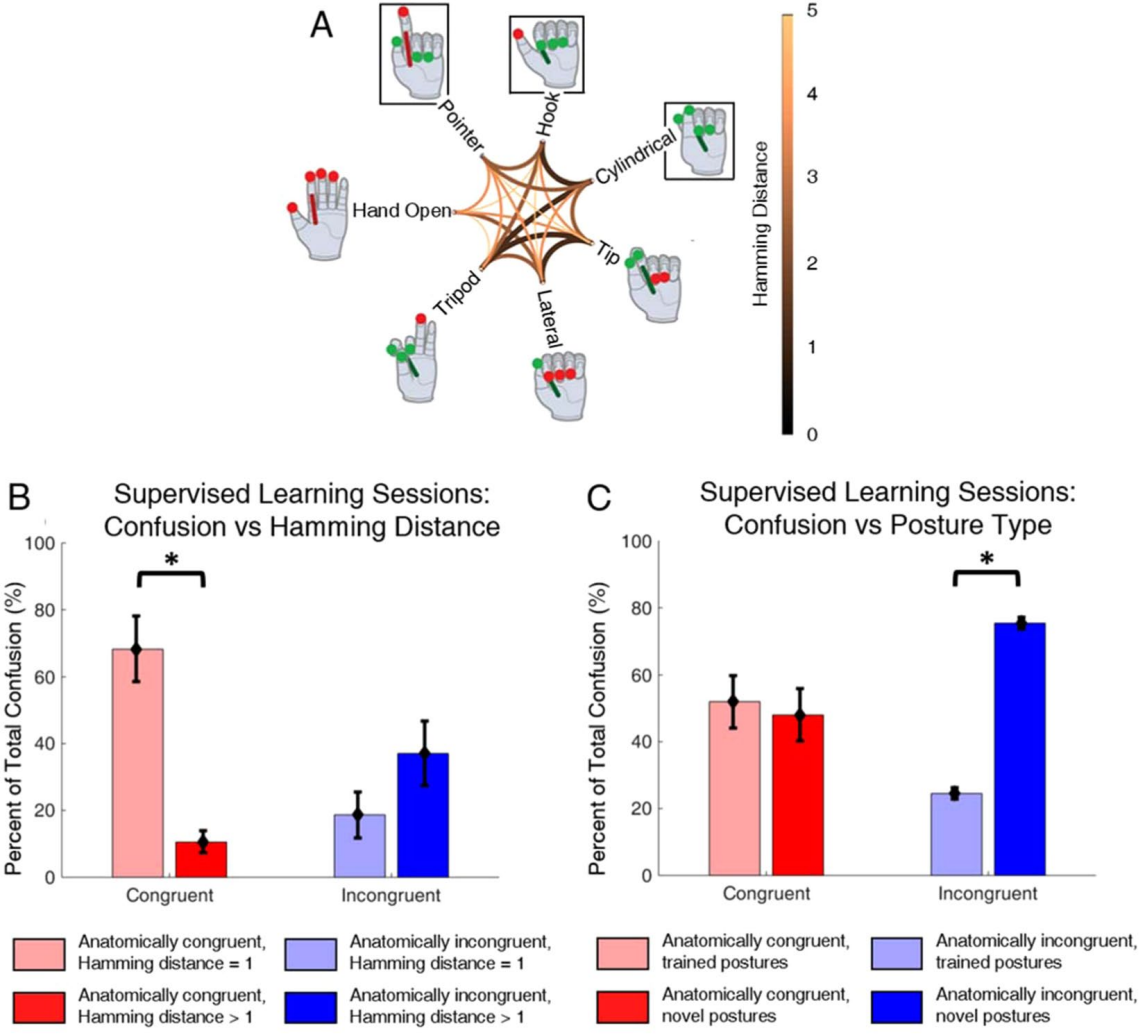
(**A**) Hamming distance between postures as a function of active sensors. Lines connecting postures become thicker and darker as the Hamming distance decreases and similarity increases. Novel postures are denoted by black boxes. (**B**) Proportion of confused posture pairs (%) whose Hamming distances were small versus large. Similar postures made up a significant portion of total confused postures pairs in the congruent mapping (n = 47, 30 misidentified postures in the congruent and incongruent mappings, respectively). (**C**) Proportion of incorrectly identified postures that were trained versus novel. The participant predominantly misidentified novel postures in the incongruent mapping (n = 47, 30 misidentified postures in the congruent and incongruent mappings, respectively).

Finally, different factors influenced the participant’s errors throughout the SL blocks between the two mappings. Two factors were investigated: similarity of posture pairs and novelty of presented posture. The influence of posture similarity on errors was analyzed using Hamming distance (Fig. 6A). Four of the twenty-one posture pairs (19%) were similar as defined by a Hamming distance of one. If the similarity of two postures did not influence the likelihood that the participant would confuse them, then we expect 19% of his errors to occur between similar postures. Alternately, we expect the percentage of total errors that occurs on presentation of novel postures to be significantly higher than 19% if similarity matters. Interestingly, the participant predominantly confused similar postures at a significantly higher rate than expected (1-sample t-test to H_0_ = 19%, p = 0.007) in the congruent mapping (Fig. 6B, red) but did not in the incongruent mapping (1-sample t-test to H_0_ = 19%, p = 0.961) (Fig. 6B, blue).

The influence of posture novelty on errors was analyzed similarly. 42.9% (30/70) of trials in each block required the participant to identify a novel posture. Training did not influence the participant’s errors in the congruent mapping (1-sample t-test to H_0_ = 42.9%, p = 0.55) (Fig. 6C, red). Conversely, posture novelty did influence his errors in the incongruent mapping (1-sample t-test to H_0_ = 42.9%, p < 0.001) (Fig. 6C, blue).

### Bayesian decision-making model explains experimental posture identification

**Figure 7:**
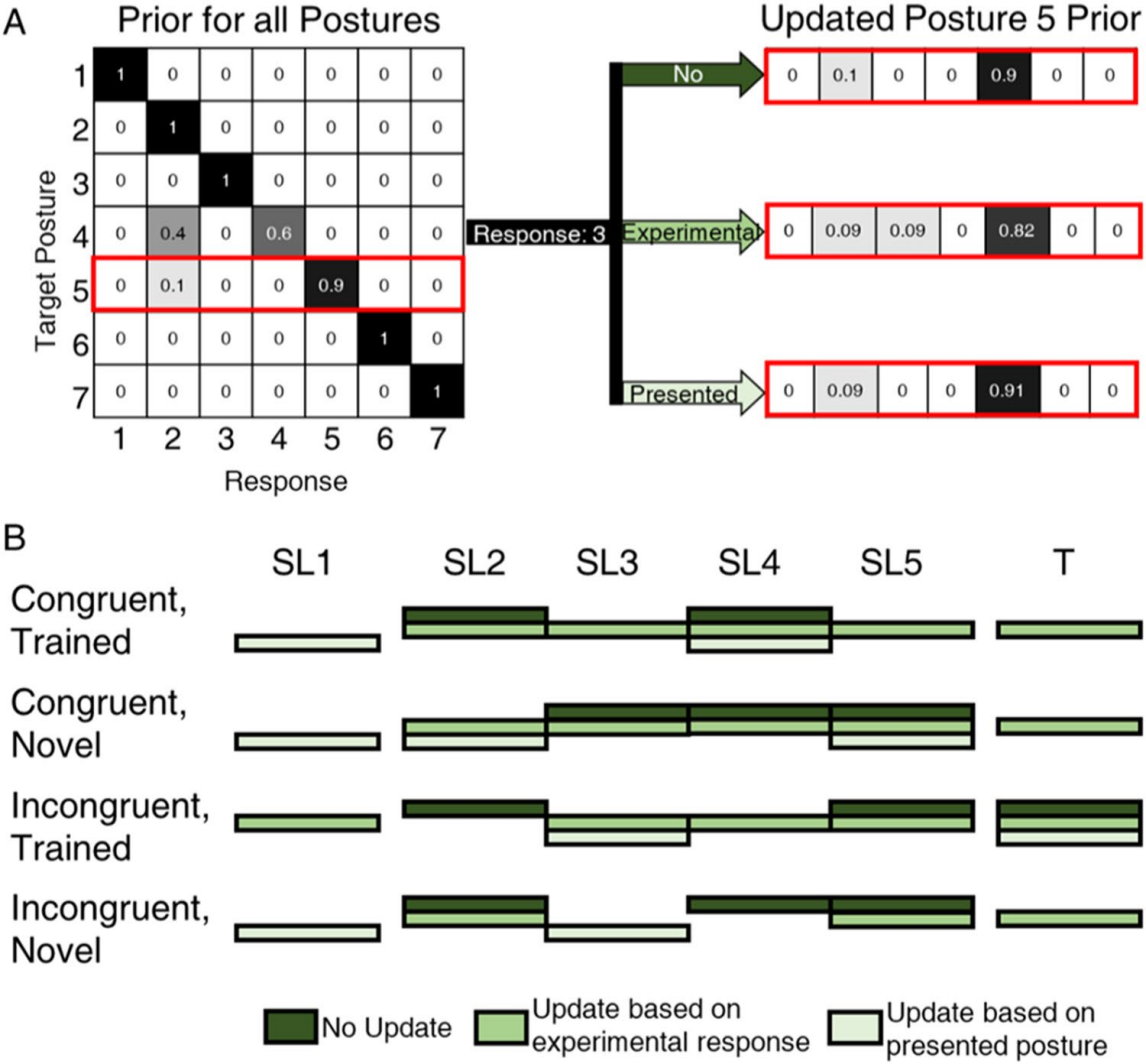
(**A**) Example of how the prior was updated in each updated method. Each row of the confusion matrix is the probability distribution function for the given posture and is defined as the prior in the Bayesian model presented here. Then, the prior is either not updated (top), updated based on the experimental response (middle) or updated based on the presented posture (bottom). (**B**) Infographic of best-fitting update methods for each of the SL and T blocks. In cases where one update method was not significantly better than the others, all methods with statistically equivalent error are presented in the infographic (n = 100 Monte Carlo iterations of each block under each update rule).

We implemented a Bayesian model of the participant’s decision-making process. The prior in the model was updated using one of three rules. The “no update” rule modeled a scenario in which the prior remained constant, indicating that the subject was not learning (Fig. 7A, top). The “experimental” rule modeled a scenario in which participant’s experimental decisions updated his prior, regardless of whether or not they were correct (Fig. 7A, middle). We hypothesize this scenario would occur if the participant’s existing associations between sensation and posture dominated the learning process, such as when no corrections were provided. In contrast, the “presented” rule modeled a scenario in which the corrections provided to the participant after each trial reinforced the correct associations between sensation and postures (Fig. 7A, bottom). Note that if the participant’s experimental performance was both high and stable, all three update rules would predict equivalent performance. “Experimental” decisions would be identical to “presented” decisions since the participant would be making correct decisions. Further, the participant would be updating his prior, so the “no update” rule would also predict correct decisions.

These Bayesian models predicted the participant’s experimental performance with an average error of 7.5%. However, the update rules which best matched the participant’s performance changed between experimental blocks (Fig. 7B), providing insight into the participant’s learning process. A detailed depiction of the percent error of each model across experimental sessions is provided in Supplemental Fig. 2. In the first supervised learning session (SL1), the “presented posture” rule fit best for three of the four conditions: congruent-trained, congruent-novel, and incongruent-novel (1-way ANOVA followed by Tukey, p < 0.001) (Fig. 7B, SL1). This indicates that the participant was utilizing the corrections provided after each trial to rapidly improve his performance on the posture identification task. The best-fitting update rule for these three conditions was more variable in the remaining SL sessions. There do not seem to be trends which distinguish a certain Bayesian prior as being the most descriptive of the participant’s behavior during the SL2-SL5 sessions. The lack of consensus across SL2-SL5 demonstrates that the participant was likely not using a consistent method to make decisions in these sessions. Finally, the “experimental update” rule fit best for the congruent-trained, congruent-novel, and incongruent-novel postures in the T block (1-way ANOVA followed by Tukey, p < 0.001) (Fig. 7B, T). Correction was not provided during the T block, which may have enabled the participant’s performance to drift as he reinforced his incorrect responses.

The Bayesian model results further corroborate that the participant engaged differently with the incongruent-trained postures as compared to the other postures (Fig. 7B, incongruent-trained postures). Given that correct, stable performance is predicted equally by all three update rules, there are no clear trends shown by the Bayesian model for the incongruent-trained postures in the SL sessions. Similarly the participant maintained high, stable performance in identifying the incongruent-trained postures in the T block, so all three models fit equally (1-way ANOVA, p = 0.429) (Fig. 7B, T).

## Discussion

In this case study, we show that five somatosensory percepts can be combined and used to identify hand postures without other sensory information and in the absence of voluntary motor control. Our results indicate that at least five spatially distinct percepts created by electrical stimulation can be successfully utilized by the participant to perform a task. This task differs from object identification tasks previously reported in the literature in which participants were asked to identify object features like size or stiffness^28,32^. In those experiments, task performance depended on perception of gradations of a single sensory percept such as aperture of the hand or intensity of tactile sensation on a fingertip. In contrast, the posture identification task reported on here required the participant to combine all provided somatosensory feedback in order to correctly identify hand postures. The participant accomplished the task by developing a holistic understanding of the provided sensory feedback for individual postures and of relationships between postures. While explicit study of the mechanism underlying the combination process is left to future work, the participant’s high accuracy in identifying postures reported in this study supports that such a process did occur.

We noted distinct differences in the participant’s ability to combine somatosensory feedback between the congruent and incongruent mappings. As expected, the congruent mapping enabled better generalization and a faster learning experience. However, the purpose of the incongruent mapping was to provide a baseline measurement of the participant’s rote memorization abilities. As the complexity and amount of information provided was constant between the two conditions, any differences between the incongruent and congruent mappings were attributed to the anatomical appropriateness of the sensation. Within this framework, we hypothesized that differing internal hand representations drove the differences between the congruent and incongruent conditions. Specifically, providing sensory feedback in a congruent manner may have allowed the participant to use his extant hand representation in developing a prediction of the prosthesis hand posture and expected interactions^75–77^. Conversely, the participant needed to either generate an entirely new hand representation or actively reason to utilize incongruent information since this mapping was incompatible with his previous experiences. Engaging the extant hand representation in the congruent mapping would improve generalization to novel stimuli^50,57,78^, as he could rely on comprehensive knowledge of all hand postures acquired over his lifetime to make inferences about novel hand postures. In contrast, the inability to utilize the extant hand representation in the incongruent mapping would force the participant to extrapolate the knowledge acquired over three experimental blocks when generalizing to novel postures, making identification of postures more error-prone^79,80^. Indeed, the participant was able to verbalize a strategy for the G block in the congruent mapping, indicating that he understood what the sensations for the novel postures should be and how the sensations differed between postures. In contrast, the participant had difficulty developing a clear strategy for identifying postures in the incongruent mapping. When asked about his performance during the generalization task with incongruent stimuli, the participant said that the task was “Terrible. I stuck with what I knew…Throw somebody to the wolves, why don’t you?” The participant waited until he was able to obtain information about the postures during the SL blocks to begin attempting to identify the novel postures. Throughout the G block of the incongruent mapping, he refused to engage with the novel postures and simply over-responded with a trained posture instead (Fig. 3A). Even after training in the SL blocks, his ability to identify the novel postures in the incongruent mapping lagged behind that of the trained postures (Fig. 5A,B).

Combination of artificial sensory feedback with the extant hand representation would also have allowed the participant to immediately focus on learning relationships between postures during the SL bocks instead of simply memorizing the provided feedback. His equivalent performance in identifying trained and novel postures in the congruent mapping (Fig. 5A,B, red) indicates that the participant was successfully able to refine his hand representation as he accumulated information throughout the SL trials. In contrast, the participant’s ability to identify novel postures was significantly lower than his ability to identify trained postures in the incongruent condition (Fig. 5A,B, blue), indicating that novel postures were not equally represented in his understanding of the sensory feedback. This is further reflected in the differing strategies he used to identify postures between the mappings. The participant’s strategy for identifying postures in the congruent mapping reflected the similarities in active sensor locations and stimulation-evoked sensory percepts across postures (Fig. 6B, red). The three postures that the participant most often confused in the congruent mapping (tip, tripod, and cylindrical) all had similar active sensor locations (Hamming distances = 1), indicating that the participant prioritized relationships between postures in identifying any single posture. Conversely, the participant’s decision-making process in the incongruent mapping de-emphasized relationships between postures (Fig. 6B, blue) and focused on rote memorization of each posture. The participant memorized the trained postures during the four-posture blocks but did not fully memorize the novel postures in the seven-posture blocks (Fig. 2C). Thus, it was the novelty of postures instead of similarity of postures that drove his errors when identifying grasps, as indicated by his consistent performance in identifying trained postures and poor performance in identifying novel postures (Fig. 5A, blue solid). The Bayesian model corroborates these experimental results and provides additional insight to the decision-making processes used by the subject throughout the experimental sessions. The Bayesian model results most clearly depict a differing strategy used by the subject when responding to the incongruent-trained postures compared to the congruent-trained, congruent-novel, and incongruent-novel postures. During the SL1 and T sessions, the incongruent trained postures were best modeled by a different Bayesian prior compared to the three other types of postures. In this way, we can distinguish how the subject was treating the incongruent trained postures as different than the others when first learning the postures (SL1) and when being tested with correction (T).

Utilization of the extant hand representation would have increased the participant’s familiarity with expected sensations. As a result, he would be able to extract information salient to the task more efficiently^38,40–43,75,77,81^ and reduce the cognitive effort associated with utilizing this information for decision-making. Response latency is often utilized as an indirect measure of cognitive effort^69–73^. Indeed, the participant’s response time was not significantly different between trained and novel postures in the congruent mapping (Fig. 5B), indicating that the participant did not find it more cognitively difficult to identify novel postures. However, he required significantly more time to identify incongruent-novel postures, suggesting that he found identification of these postures more cognitively demanding (Fig. 5B).

Providing sensory feedback through a congruent mapping may increase the functionality and ease-of-use of a sensory prosthesis system, two critical determinants of choosing to wear a prosthetic device or not^22–25^. However, it is encouraging that the participant was still able to effectively use incongruently provided feedback in simple tasks or after training. Indeed, in the four-posture task, the participant could fully compensate for the incongruent nature of the somatosensory stimuli and achieved nearly perfect posture identification (Fig. 2A,C). He was also able to significantly improve his ability to identify all seven postures with training (Fig. 4A). These improvements occurred within a single ten-block experimental session. The remaining differences between congruent and incongruent performance may be eliminated after prolonged exposure to an incongruent mapping, as would occur when utilizing the device as part of one’s daily routine over weeks or years. As such, we show that incongruent feedback still provides useful sensory information. This can be of importance in situations where providing congruent feedback is difficult, such as when an electrode fails, thereby limiting the ability to physically interact with the appropriate neural populations, or when the end effector is not anthropomorphic – for instance, there is no “index finger” on a greifer prosthesis.

In addition to simulating different methods of providing sensory feedback, we also simulated different situations in which prosthesis users may receive sensory feedback. The task, combination of simultaneous sensory feedback, is necessary for many activities of daily living. Further, the different block types in the study design (G, SL, versus T) represent different real-world scenarios. The T block lacked any corrective feedback and is most consistent with functional testing formats in the laboratory settings. The participant performed comparably between the congruent and incongruent mappings in these blocks. In contrast, the SL and G blocks better simulate usage of a sensory-enabled prosthesis in real-world settings. Prosthesis users receive instantaneous feedback on the successfulness of task performance by comparing their knowledge of current performance to their expected performance. For example, when performing a cylindrical grasp, the user is immediately aware of whether they successfully achieved the posture or not. The SL blocks mimicked this real-time feedback by providing correction after each trial. Further, daily usage occurs in an unconstrained environment, and prosthesis users will inevitably encounter novel scenarios. By introducing three novel postures, the G block models how a user might react to novel sensory feedback. In both the SL and G blocks, the participant performed differently between the congruent and incongruent mappings, suggesting he might utilize and incorporate provided somatosensory information differently when performing in activities of daily living as well. We conclude that information should be presented in a congruent manner when possible, reserving the use of incongruent stimuli for use only when necessary due to technical limitations.

This work has several limitations. As this is a case study, it is difficult to expand the results interpretation to the general amputee population. The peripheral nerve interface used here can elicit percepts across the hand in both tactile and proprioceptive modalities, but the spatial accuracy and coverage is not identical to that of the intact somatosensory system. Because of this, the proprioceptive sensation, while congruent with natural proprioception in modality, was spatially incongruent. Interestingly, the participant had higher accuracy in identifying postures that did not include proprioceptive feedback, suggesting that his mechanism for combining somatosensory inputs varied depending on whether all percepts were cutaneous or were mixed cutaneous and proprioceptive. An alternative explanation is that the spatial incongruence of the proprioceptive feedback in the anatomically congruent condition may account for these changes in accuracy. Future work will attempt to resolve this question by utilizing spatially congruent proprioceptive sensation in this task. Another limitation is that only five somatosensory percepts were provided to the participant, and the percepts did not fully cover the hand. The maximum number of discernable and differentiable percepts from peripheral nerve stimulation was not quantified in this study, but is an interesting area for future research. Finally, the fidelity of the sensations provided was not explicitly studied, so further study into the number and fidelity of sensory percepts necessary to provide a complete hand representation is necessary.

Here, we show that a prosthesis user was able to combine five artificial somatosensory percepts to identify prosthesis hand postures. Further, utilizing Bayesian modeling and information theory approaches, we found that his strategy, ability to generalize, and ability to combine information depended on the anatomical congruency of the mapping used. While our ability to expand this case study’s results to a larger population is limited, we show that this single participant was able to achieve better outcomes with less cognitive effort when presented with congruent stimuli as compared to incongruent stimuli.

## Supplementary information

Supplementary information
