# Supplementary material for: Combination of Simultaneous Artificial Sensory Percepts to Identify Prosthetic Hand Postures: A Case Study
Source: Sci Rep. 2020 Apr 20;10:6576. doi: 10.1038/s41598-020-62970-4 (PMC7171192; doi:10.1038/s41598-020-62970-4)
Supplement: Supplementary file 1 — Supplementary information [file 41598_2020_62970_MOESM1_ESM.docx]

Supplemental Information

Combination of Simultaneous Artificial Sensory Percepts to Identify Prosthetic Hand Postures: A Case Study

Jacob L. Segil1,2†, Ivana Cuberovic3,4†, Emily L. Graczyk3,4, Richard F. *ff.* Weir1,5, and Dustin Tyler3,4, *

1 Rocky Mountain Regional VA Medical Center, Rehabilitation Research and Development, Denver, CO, 80220, USA

^2^ University of Colorado Boulder, Engineering Plus Program, Boulder, CO 80309, USA

3Case Western Reserve University, Department of Biomedical Engineering, Cleveland OH, 44106, USA

^4^ Louis Stokes Cleveland Veterans Affairs Medical Center, Cleveland, OH, 44106, USA

^5^ University of Colorado Denver | Anschutz Medical Campus, Department of Bioengineering, Aurora, CO, 80045, USA

* dustin.tyler@case.edu

†Both authors contributed equally to this work

**Supplemental Table 1**: Reported percept locations for each sensor. Asterisk indicates a proprioceptive sensation in which the participant feels flexion of the phantom ring finger. The participant reported the location of the perceived sensation at the experimental stimulation parameters multiple times. The frequency with which each pixel is reported is indicated by increasing color opacity.

|  | Congruent | | Incongruent | |
| --- | --- | --- | --- | --- |
| Sensor Location | Stimulation Location | Evoked Sensory Location | Stimulation Location | Evoked Sensory Location |
| Thumb | Thumb | 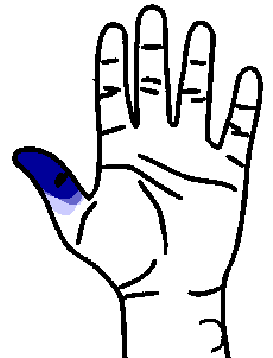 | Index | 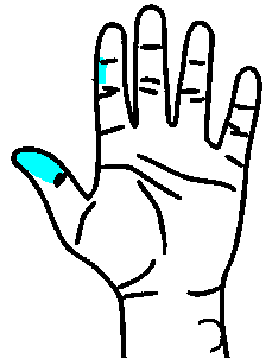 |
| Index | Index | 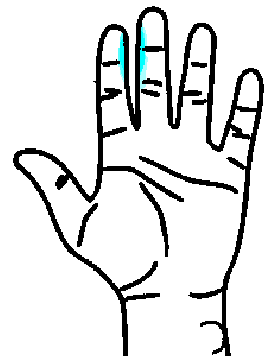 | Ring/ Pinkie | 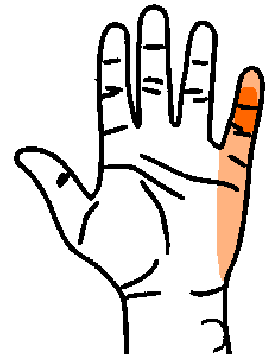 |
| Middle | Middle | 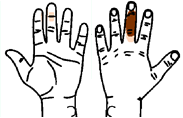 | Flexion | 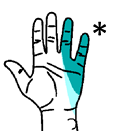 |
| Ring/ Pinkie | Ring/ Pinkie | 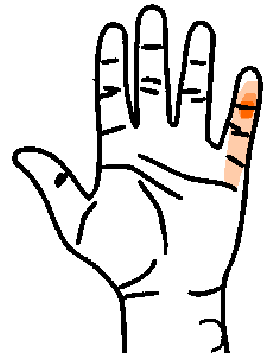 | Middle | 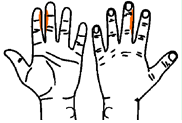 |
| Index Flexion | Flexion | 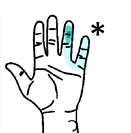 | Thumb | 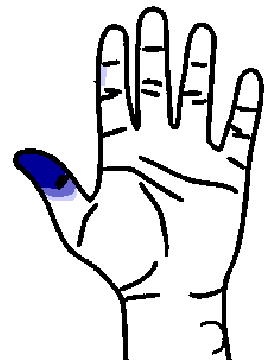 |


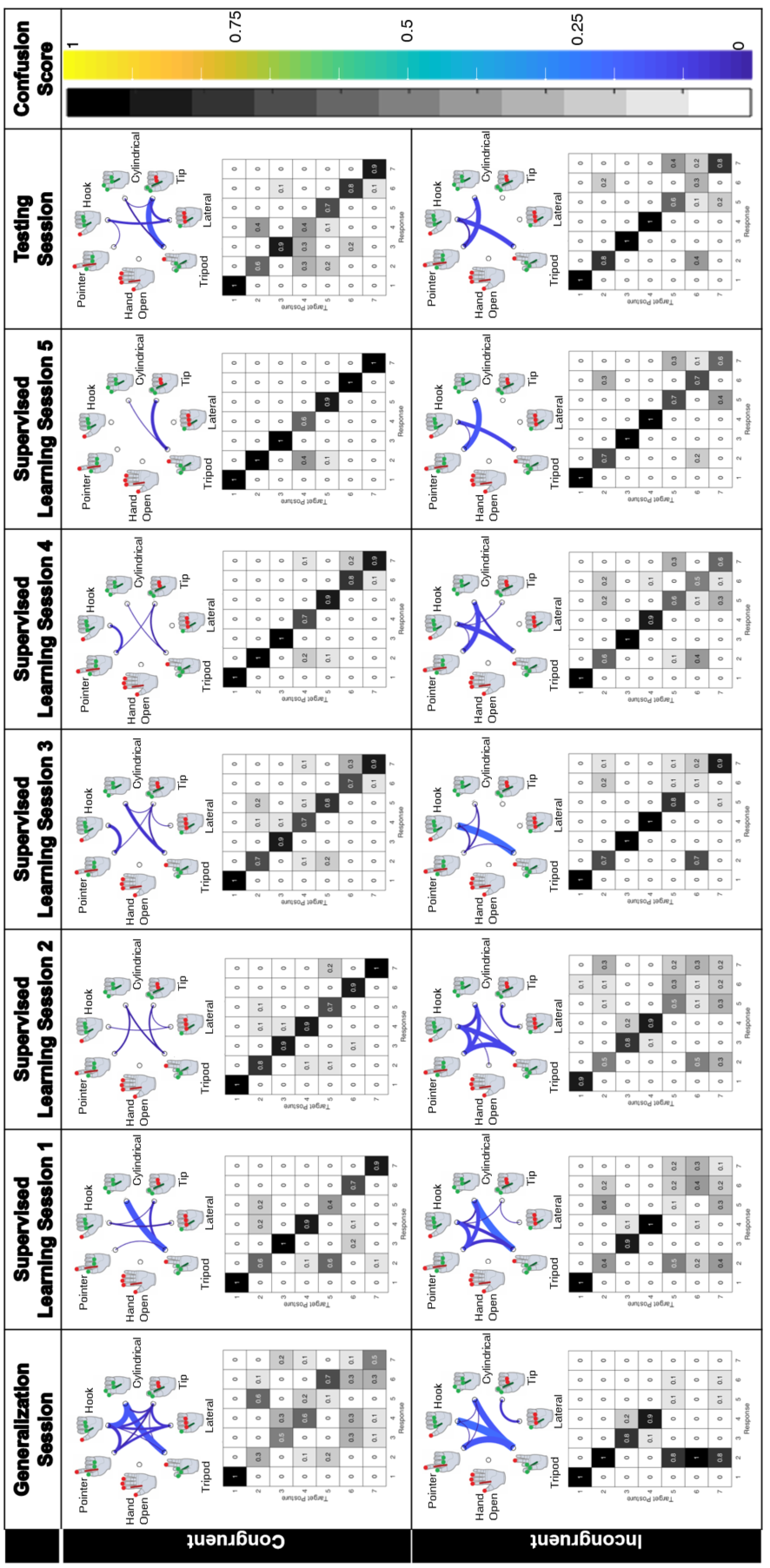


**Supplemental Figure 1**: Chord diagrams and associated confusion matrices for the seven-posture identification blocks (columns) for the congruent (top) and incongruent (incongruent) mappings. Confusion decreases over the blocks. Within a block, there is more confusion in the incongruent mapping than in the congruent mapping (n = 10 presentations of each posture per block).


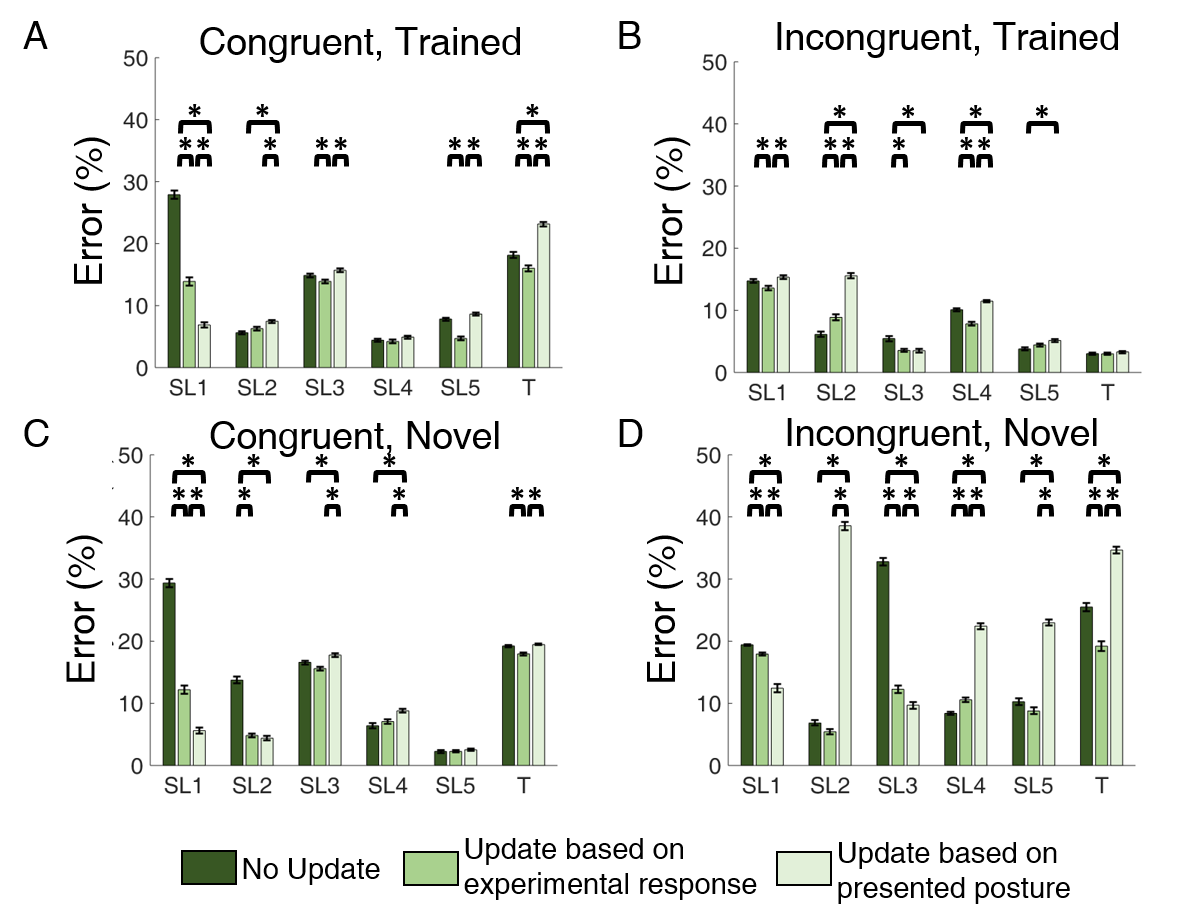


**Supplemental Figure 2**: The error between the Bayesian model accuracy and the empirical accuracy using three methods of updating the Bayesian prior are depicted. The relative error between models is presented for the congruent trained postures (**a**), incongruent trained postures (**b**), congruent novel postures (**c**), and incongruent novel postures (**d**). All significance bars indicate p ≤ 0.001 for a 1-way ANOVA followed by Tukey test (n = 100 Monte Carlo iterations of each block under each update rule).
